# TRESK channel contribution to nociceptive sensory neurons excitability: modulation by nerve injury

**DOI:** 10.1186/1744-8069-7-30

**Published:** 2011-04-28

**Authors:** Astrid Tulleuda, Barbara Cokic, Gerard Callejo, Barbara Saiani, Jordi Serra, Xavier Gasull

**Affiliations:** 1 Neurophysiology Lab, Dept. Physiological Sciences I, Medical School, University of Barcelona - Institut d'Investigacions Biomèdiques August Pi i Sunyer (IDIBAPS), Barcelona, Spain; 2 Neuroscience Technologies, Barcelona Science Park, Spain

## Abstract

**Background:**

Neuronal hyperexcitability is a crucial phenomenon underlying spontaneous and evoked pain. In invertebrate nociceptors, the S-type leak K^+ ^channel (analogous to TREK-1 in mammals) plays a critical role of in determining neuronal excitability following nerve injury. Few data are available on the role of leak K_2P _channels after peripheral axotomy in mammals.

**Results:**

Here we describe that rat sciatic nerve axotomy induces hyperexcitability of L4-L5 DRG sensory neurons and decreases TRESK (K2P18.1) expression, a channel with a major contribution to total leak current in DRGs. While the expression of other channels from the same family did not significantly change, injury markers ATF3 and Cacna2d1 were highly upregulated. Similarly, acute sensory neuron dissociation (*in vitro *axotomy) produced marked hyperexcitability and similar total background currents compared with neurons injured *in vivo*. In addition, the sanshool derivative IBA, which blocked TRESK currents in transfected HEK293 cells and DRGs, increased intracellular calcium in 49% of DRG neurons in culture. Most IBA-responding neurons (71%) also responded to the TRPV1 agonist capsaicin, indicating that they were nociceptors. Additional evidence of a biological role of TRESK channels was provided by behavioral evidence of pain (flinching and licking), in vivo electrophysiological evidence of C-nociceptor activation following IBA injection in the rat hindpaw, and increased sensitivity to painful pressure after TRESK knockdown in vivo.

**Conclusions:**

In summary, our results clearly support an important role of TRESK channels in determining neuronal excitability in specific DRG neurons subpopulations, and show that axonal injury down-regulates TRESK channels, therefore contributing to neuronal hyperexcitability.

## Background

After peripheral axon injury, nociceptors undergo a variety of changes resulting in persistent hyperexcitability and ectopic discharge, all potentially leading to altered pain perception, such as spontaneous pain, hyperalgesia and allodynia [1,2]. Constricting lesions and partial or total axotomy of peripheral nerves in animals produce behavioral alterations analogous to those seen in human neuropathic pain [3,4]. After injury to peripheral branches of nociceptors due to trauma, inflammation or other noxious stimuli, a variety of post-translational and transcriptional changes modifies nociceptor normal function [5] leading to abnormal sensory transduction and persistent hyperexcitability that contribute decisively to neuropathic pain. Change in the expression levels and/or biophysical properties of ion channels, receptors, growth factors and neuropeptides contribute to increased input resistance (R_in_), decreased action potential (AP) threshold and accommodation, and to the presence of postdischarge and ectopic activity in nociceptors [6,7].

In invertebrate and mammalian sensory neurons, hyperexcitability is expressed as a decreased spike threshold and/or repetitive firing during prolonged depolarizing stimuli [7-11]. A common finding in injured neurons is an increased R_in_, which reflects a decrease in membrane conductances active at/or near resting potential and facilitates reaching AP threshold. Most studies in sensory neurons have focused in voltage-dependent ion channels that shape AP and contribute to cellular excitability. Less attention has been given to *leak *K^+ ^channels, despite their role in setting membrane excitability [12-15]. Several background K^+ ^channels from the K_2P _family, including TREK-1 and -2, TASK-1, -2 and -3, TRAAK and TRESK, are expressed in DRG and trigeminal neurons, [16-18]. In small and medium-sized DRGs, major background currents are carried by TREK-2 and TRESK while smaller contributions were encountered for TREK-1 and TRAAK [19]. Despite the latter, TREK-1 is involved in pain perception, as TREK-1 knockout mice show higher sensitivity to low threshold mechanical stimuli and increased thermal and mechanical hyperalgesia after inflammation [20,21]. TRESK likely contribute to membrane excitability, since TRESK[G339R] functional knockout mice shows enhanced DRG excitability [18]. A recent report links a dominant-negative mutation in hTRESK to familial migraine with aura, implicating this channel in the generation of aura pathogenesis [22]. In addition, pungent agents from Szechuan peppers (hydroxy-α-sanshool) block some K_2P _channels (TASK-1, TASK-3 and TRESK), activating sensory neurons expressing these channels [23]. Application of hydroxy-α-sanshool to sensory neuron peripheral terminals activates rapidly and slowly adapting Aβ fibers, rapidly adapting D-hair fibers (Aδ) and a subset of slowly conducting C fibers [24]. Similarly, the synthetic alkylamide IBA activates low-threshold mechanosensitive and wide-dynamic range spinal neurons that receive convergent input from mechanoreceptors and nociceptors [25]. Here we show that the background channel TRESK, is down regulated in a model of neuropathic pain, which likely contributes neuronal hyperexcitability induced by nerve injury. Also, blocking or silencing the channel produces activation of sensory neurons and nociceptive fibers as well as behavioral evidence of pain.

## Results

### Axotomizing injury decreases TRESK channels expression

**Figure 1:**
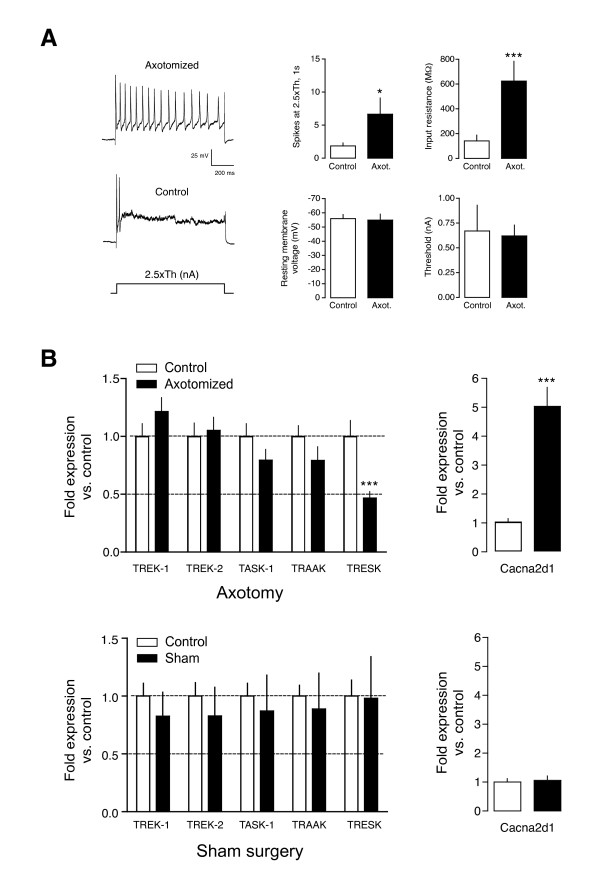
**Axotomy effects on background K^+ ^channels**. **A**. Sciatic nerve axotomy induces hyperexcitability of DRG sensory neurons recorded in the acutely dissected ganglion. Excitability was measured as the number of spikes fired by a 1s pulse 2.5× action potential threshold. *Left*. Examples of an axotomized and a contralateral uninjured control neuron. *Right: *Quantification of number of spikes fired, input resistance, resting membrane potential and action potential threshold. *p < 0.05; ***p < 0.001 t-test axotomized vs. control (n = 8 for each group). **B**. Effect of axotomy (top) and sham surgery (bottom) on K_2P _channels mRNA. Expression changes were normalized with the contralateral uninjured side for each independent animal (n = 4) and expressed as mean ± SEM. A significant decrease in expression was found for TRESK (***p < 0.001). As a positive control, expression of the calcium subunit Cacna2d1 was detected and found upregulated after axotomy (p < 0.001), as previously described, but not after sham surgery.

It is long known that peripheral axon injury produces sensory neuron hyperecitability [7,26]. In order to study changes in background conductances contributing to this phenomena, we recorded a few neurons in L4-L5 DRG to confirm that they were hyperexcitable 3 weeks after sciatic nerve axotomy. Intracellular recordings in small-medium DRG neurons (soma: 26.6 ± 0.6 μm; range 22-30) in the excised intact ganglia showed an increased soma excitability measured as the number of axon potentials fired by a normalized 1-s depolarizing pulse 2.5× the 20 ms spike threshold current. Axotomized neurons fired more spikes (6.5 ± 2.4 spikes; mean ± SEM; n = 8) compared with contralateral uninjured neurons (1.9 ± 0.5 spikes; n = 8; p < 0.05, unpaired t-test; Figure 1A). In addition to the increased excitability, injured neurons had a higher R_in _(624.5 ± 159.7 vs. 141.4 ± 47.8 MΩ; p < 0.001, unpaired t-test), without significant differences in resting membrane potential (RMP -54.9 ± 4.1 vs. - 55.9 ± 2.9 mV) or action potential threshold (0.62 ± 0.1 vs. 0.67 ± 0.3 nA). These results are in general agreement with previous reports of excitability of injured and uninjured sensory neurons in DRG ganglia [7,10,27,28]. In parallel, we studied whether neuronal hyperexcitability produced by injury can be due to gene expression changes in background K_2P _channels that are known to be expressed in DRG neurons [17,29]. Injured DRG neurons did not show significant changes in the transcripts for TREK-1, TREK-2, TASK-1 or TRAAK (1.22 ± 0.23; 1.05 ± 0.07; 0.80 ± 0.06; 0.79 ± 0.23 fold-change, respectively) but displayed a significant reduction in TRESK transcripts (0.46 ± 0.07 vs. contralateral side; p < 0.001; Figure 1B; n = 4 independent animals). As previously described, injury increases the expression of the α2δ1 subunit of the voltage-dependent calcium channel [30], thus we used its expression as a positive control. In the same samples, the transcript for this subunit was increased by 5-fold when compared to the contralateral uninjured side (Cacna2d1: 5.00 ± 0.47; p < 0.001; Figure 1B). Sham surgery did not show significant changes in the expression of any of the tested genes when compared to the contralateral uninjured side (TREK-1: 0.83 ± 0.21; TREK-2: 0.83 ± 0.24; TASK-1: 0.87 ± 0.31; TRAAK: 0.89 ± 0.31; TRESK: 0.98 ± 0.36; Cacna2d1: 1.06 ± 0.14; n = 4; Figure 1B). When axotomized and sham animal groups where compared, only TRESK (p < 0.05) and Cacna2d1 (p < 0.001) showed significant differences on channel expression. Changes in opposing directions were seen when comparing the expression of TREK-1 in axotomized and sham animals (Figure 1B) that were close but not statistically significant (p = 0.09).

**Figure 2:**
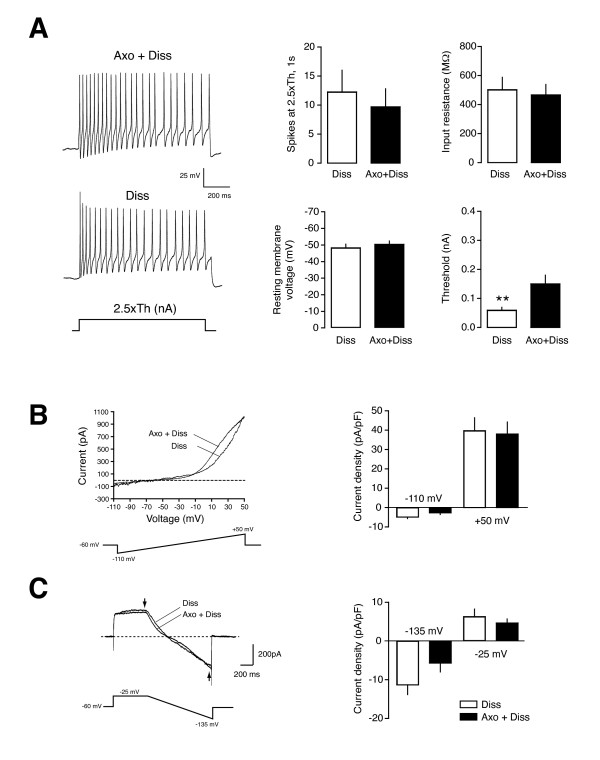
**In vitro and axotomy produces similar changes than in vivo axotomy**. **A**. Acute dissociation of DRG sensory neurons produces similar hyperexcitability in previously injured or non-injured neurons. *Left: *Examples of spikes fired by a neuron axotomized in the animal and later dissociated and another cell only acutely dissociated. Right: Quantification of number of spikes fired, input resistance, resting membrane potential and action potential threshold. **p < 0.01 t-test Dissociated (Diss; n = 16) vs. Axotomized+dissociated (Axo+Diss; n = 15) cells. **B**. Effects of in vitro axotomy in total whole-cell currents. Currents elicited by a voltage ramp (-110 to +50 mV) in Diss. and Diss+Axo neurons (n = 7 for each group) in the presence of 2 μM TTX. Quantification did not show significant differences in currents at -110 or +50 mV. **C**. Examples of recordings in Diss. and Diss+Axo neurons using a protocol to minimize activation of voltage-gated transient K^+ ^outward currents. Quantification of currents at the end of the pulse at -35 mV (arrow) and at the end of the ramp (-135 mV; arrow) did not show significant differences among groups.

To further investigate the effect of injury on background channels, we compared whether "*in vitro *axotomy" produced by dissociation of DRG neurons rendered similar effects than *in vivo *axotomy (sciatic nerve transection). Despite the fact that dissociated DRGs may have several differences with neurons axotomized in the animal, others have reported that dissociation of DRGs produces neuronal hyperexcitability [11,31], thus we asked whether similar changes underlie this hyperexcitable state. As expected, small and medium-sized neurons (soma: 23.2 ± 0.5 μm; range 16-27) axotomized in the animal and then dissociated (Axo+Diss group) were hyperexcitable (9.7 ± 3.1 spikes; n = 15; Figure 2A). However, uninjured but dissociated neurons (Diss group), also showed marked hyperexcitability (12.3 ± 3.7 spikes, n = 16), confirming that neuronal dissociation alone also induces a significant increase in cell excitability. No significant differences were obtained for R_in_, RMP, AP amplitude, AP duration or AHP amplitude, although Diss neurons had a slightly lower action potential threshold compared with neurons in the Axo+Diss group (0.06 ± 0.01 vs. 0.15 ± 0.03 nA; p < 0.01; Figure 2A). To assess whether injury modifies total background currents in DRG neurons, we performed whole-cell patch clamp recordings in both groups of neurons using a ramp protocol and in the presence of 2 μM TTX. Current measurements at -110 mV (Axo+Diss: -2.7 ± 0.9 pA/pF; Diss: -4.8 ± 0.7 pA/pF; n = 7; Figure 2B) or at +50 mV (38.1 ± 6.2 and 39.7 ± 6.8 pA/pF; n = 7) did not show significant differences between groups. In addition, using a protocol to minimize activation of voltage-gated transient K^+ ^outward currents [18], outward and inward currents measured at the end of the depolarizing step and the hyperpolarizing ramp (arrows; Figure 2C) also failed to elicit significant differences between Axo+Diss (-5.7 ± 2.3 pA/pF at -25 mV and 4.6 ± 1.0 at -135 mV; n = 7) and Diss neurons (-11.3 ± 2.5 pA/pF at -25 mV and 6.2 ± 1.9 pA/pF at - 135 mV; n = 5), despite the fact that Diss neurons appeared to have a slightly higher outward current. Because patch clamp recordings were done at room temperature (~22°C) in small- and medium-sized neurons were TRESK is preferentially and abundantly expressed [18,29] and due to the fact that other background channels present in DRGs are mostly inactivated at this temperature [19,32], the recorded background currents should be mainly carried by TRESK channels.

**Figure 3:**
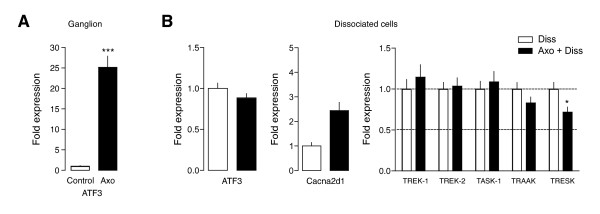
**Positive injury markers in dissociated and axotomized neurons**. **A**. Upregulation of injury marker ATF3 in neurons axotomized in vivo (***p < 0.001; n = 4 animals). **B**. ATF3 shows similar levels of expression in Diss. and Diss+Axo neurons (n = 3 independent dissociated cultures). Notice that a similar fold change is obtained when normalizing with Diss. expression levels, implying that expression levels of Diss. neurons had increased to levels in axotomized neurons. Cacna2d1 and TRESK expression also followed the same pattern (compare data in Fig 3B and Fig 1B) although a significant decrease in TRESK is still seen in Diss+Axo neurons compared to Diss. neurons only (*p < 0.05; n = 4 independent experiments).

To correlate the effects of injury with the expression of background channels, we next tested an injury marker described to be highly up-regulated in DRG neurons [33-37]. In *in vivo *axotomized neurons, ATF3 expression was increased by 25-fold compared with contralateral uninjured cells (Figure 3A; p < 0.001; n = 4 animals). In contrast, ATF3 expression did not show significant differences between Axo+Diss and Diss neurons (Figure 3B; n = 3 independent dissociated cultures), meaning that this transcript was now upregulated in both groups. Similarly, the differential expression of Cacna2d1previously found in the ganglia (Figure 1B) was greatly reduced, with no significant differences between groups (compare data on Figure 1B and 3B). In a similar fashion, the reduction in TRESK expression found *in vivo *was greatly diminished in the dissociated neurons, but a still significantly decreased expression was found between the Axo+Diss and the Diss group (0.72 ± 0.06; n = 4; p < 0.05; Figure 3B). All together, this data suggest that neuron dissociation produces similar changes to those caused by directly injuring the cells in the living animal, as clearly shown by the expression of injury markers Cacna2d1 and ATF3, and confirm downregulation of TRESK channel expression by nerve injury.

### Participation of TRESK channels in sensory neuron excitability

**Figure 4:**
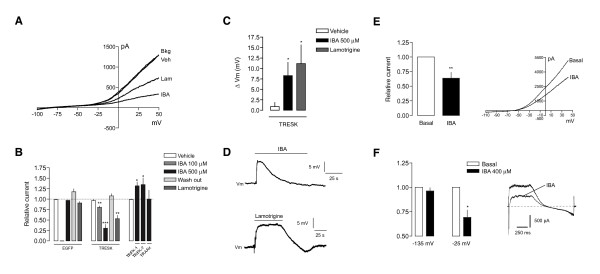
**Alkylamides block TRESK channel currents**. **A**. Representative recording of IBA (500 μM) or Lamotrigine (Lam; 100 μM) -evoked inhibition of TRESK in HEK293 transfected cells. Total background currents (Bkg) were recorded in the whole cell configuration of the patch clamp technique (Holding potential = -60 mV). Vehicle (Veh) did not produce significant effects on recorded currents. **B**. Quantification of IBA and Lam effects on TRESK and other K_2P _channels. n = 5-7 cells per condition. *p < 0.05; **p < 0.01; ***p < 0.001. t-test vs. resting background currents. **C**. Quantification of IBA and Lam effects on resting membrane voltage in HEK293 cells transfected with TRESK (n = 4 cells per condition). Membrane voltage was normalized with resting membrane. After achieving the whole cell configuration, the patch amplifier was switched to the current-clamp mode and resting membrane voltage was recorded. *p < 0.05 t-test vs. vehicle application **D**. Representative membrane voltage recordings of TRESK-transfected cells stimulated with IBA (500 μM) or Lamotrigine (100 μM). **E**. Effect of IBA (400 μM) on total current from DRG neurons. A voltage ramp protocol from -110 to +50 mV was used. A significant decrease (p < 0.01; n = 6) in total current is seen after IBA application compared to Basal current (before IBA application). **F**. Effect of IBA (400 μM) on currents from DRG neurons using a protocol composed by a depolarizing pulse to -25 mV (holding voltage -60 mV) followed by a hyperpolarizing ramp to -135 mV (as in Fig 2). Quantification of currents is shown at -25 mV (measured at the end of the pulse) and -135 mV. *p < 0.05 vs. basal current. A representative recording is shown. Some TTX-resistant sodium currents can still be observed.

To determine the participation of TRESK in the excitability of sensory neurons, we took advantage of the reported blocking activity of alkylamides like hydroxy-α-sanshool on this channel [23,24]. IBA, an alkylamide derivative [25,38] was first tested on HEK293 cells co-transfected with rat TRESK and eGFP to verify its blocking activity on this channel. 100 μM IBA application blocked total background current by 19.6 ± 3.3% (p < 0.01; n = 6) and 69.5 ± 8.7% at 500 μM (p < 0.001; n = 7; Figure 4A, B), which returned to basal values after washing the drug. IBA did not show significant effects in control cells transfected with eGFP (Figure 4B). As previously described [39], lamotrigine 100 μM also blocked TRESK-mediated background currents by 46.9 ± 8.1% (p < 0.01; n = 6). In addition, IBA modified resting membrane voltage of TRESK transfected HEK cells (Figure 4C, D). IBA application produced a mean depolarization of 8.3 ± 3.2 mV (p < 0.05 vs. vehicle; n = 4) that returned to RMP after several seconds (Figure 4D). In a similar way, lamotrigine induced a transient depolarization of 11.2 ± 4.5 mV (p < 0.05; n = 4). We also tested the effect of IBA on the other major K_2P _channels expressed in DRGs. At the maximum concentration assayed (500 μM), IBA produced an increase in TREK-1 and TREK-2 currents (32.1 ± 9.4% and 34.8 ± 15.8% respectively; p < 0.05; n = 5; Figure 4B) and did not show significant effects on TRAAK (0.33 ± 21.1%; n = 4).

To further explore these effects, IBA was tested on currents recorded from DRG neurons in culture. Total current was recorded in the presence of TTX and using a ramp protocol (as in Figure 2B). As shown in Figure 4E, bath application of 400 μM IBA blocked part of the total current (36.6 ± 10.1% at +45 mV; n = 6; p < 0.01 vs. basal current). The effect of this compound was also tested using a protocol as in Figure 2C. IBA blocked part of the current elicited by a depolarizing pulse to -25 mV (30.8 ± 7.5%; n = 6; p < 0.05 vs. basal current; Figure 4F) and induced a small but non-significant decrease at -135 mV (3.8 ± 3%; n = 6). In summary, experiments show that TRESK-mediated background currents are transiently blocked by IBA in a dose-dependent manner. In DRG neurons, native currents are also blocked by IBA, despite a possible potentiation of other K_2P _channels. This suggests that the blocking effect of IBA on TRESK is more important than the activation of other channels, or that TRESK has a higher contribution to the background currents in DRGs.

### IBA activates nociceptive neurons in vitro

**Figure 5:**
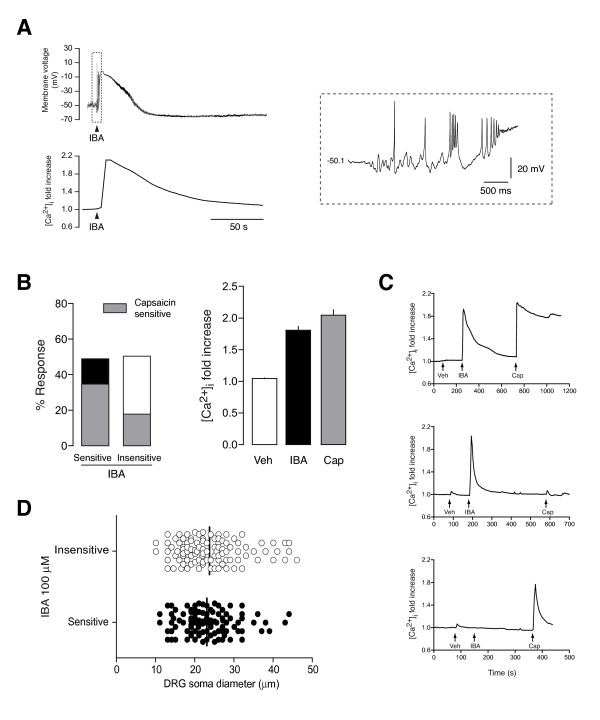
**Alkylamide effects on DRG sensory neurons**. **A**. Simultaneous measurement of membrane voltage (top) and intracellular calcium (bottom) in a neuron stimulated with IBA (100 μM). Inset: magnification of the action potentials elicited by IBA application. **B**. Quantification of intracellular calcium responses to IBA (100 μM) application in DRG neurons in culture (n = 101). *Left*. Percentage of neurons sensitive or insensitive to IBA application. In each group, the percentage of neurons responding also to capsaicin (Cap; 1 μM) is shown. *Right*. Intracellular calcium increase (fold-increase vs. resting calcium) elicited by vehicle (Veh) application, IBA (100 μM) or capsaicin (1 μM). **C**. Representative recordings of intracellular calcium in a neuron responding to IBA and Cap (top), only to IBA (middle) or only to Cap (bottom). **D**. Soma diameter distribution of DRG neurons sensitive and insensitive to IBA. Black bar shows the mean soma value in each group.

We next tested whether IBA applied to cultured DRG neurons increased intracellular calcium by inhibiting K_2P _channels, as demonstrated for hydroxy-α-sanshool [23,24]. Simultaneous recording of membrane voltage and intracellular Ca^2+ ^in DRG neurons showed that IBA application (100 μM) produces a transient membrane voltage depolarization (40.06 ± 4.16 mV) in 8 of 11 neurons tested (RMP -48.8 ± 1.5 mV) accompanied by an increase in intracellular Ca^2+ ^(1.79 ± 0.14 Fluorescence ratio R/Ro; Figure 5A). Action potential firing was also visible in these neurons upon membrane voltage depolarization (8.5 ± 3.3 spikes; Figure 5A, inset). Three neurons did not modify their membrane voltage or intracellular calcium in response to IBA application. These results confirm that inhibition of K_2P _by IBA depolarizes membrane potential and activates Ca^2+ ^entry through voltage-dependent Ca^2+ ^channels. Intracellular calcium recordings showed that 100 μM IBA activated 49.3% of DRG neurons with a mean Ca^2+ ^peak of 1.81 ± 0.06 (n = 101), while the vehicle used to dissolve the drug did not produce significant effects (Figure 5B and 5C). Among the neurons sensitive to IBA, 71.1% also responded to 1 μM capsaicin (R/Ro: 2.05 ± 0.09; n = 57), thus, as illustrated in experiments shown in Figure 5C, we identified three different subsets of neurons: 1) those selectively responding to IBA, 2) those responding only to capsaicin and 3) neurons that responded to both compounds. IBA-sensitive and non-sensitive DRG neurons showed a similar soma distribution (Figure 5D). 81.1% of the IBA-sensitive cells had soma diameters smaller than 30 μm, corresponding to small- and medium-sized DRG neurons. In the IBA-insensitive group, 77.9% of the neurons had somas <30 μm. According to this, 55.8% of small- and medium-sized neurons (<30 μm; 63/113 neurons) responded to IBA, while in large diameter neurons, the percentage of response to IBA was 41.9% (13/31 neurons). In summary, these data show that an important subpopulation of IBA-sensitive neurons are small diameter neurons (mainly nociceptors), most of them expressing TRPV1 (~70%) but not all (~30%).

### Microneurographic recordings of C-nociceptors in response to IBA injection

**Figure 6:**
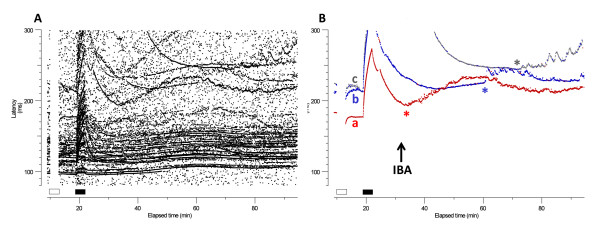
**Activation of C-fibres by IBA**. Raster plot of latencies and identification of single action potentials. **A**: Raster plot of latencies in the C-fiber range corresponding to several C fibers during baseline stimulation (0.25 Hz), pause (0 Hz, open bar) and tetanus (2 Hz, filled bar). Different fibers with particular activity-dependent profiles can be observed. **B**: Modified raster plot from A with extraction of 3 units of interest at latencies of 175, 215 and 220 ms during the period just before the 2 Hz stimulation (a, in red, CV = 0.45; b, in blue, CV = 0.32 m/s; c, in grey, CV = 0.31 m/s; CV: conduction velocity), for a better visualization. These three units displayed appreciable slowing (>2%) following the pause (open bar), and slowed more than 50% during the 2 Hz tetanus (units b and c slow beyond the 300 ms latency limit of the recorded signal), indicating they were Type 1B mechano-insensitive C-nociceptors [40]. Injection of IBA (1%, 2 μl) s.c. induced persistent ongoing discharges in all three mechano-insensitive C-nociceptors, giving rise to jittery baselines with a "saw-tooth" profile, lasting many minutes (>30 min for unit a). Onset of action was different for the three units (marked with a colored asterisk).

A total of 45 C-nociceptor units with good signal to noise ratio were recorded from the sciatic nerve of 6 rats. Of these, 24 were classified as mechano-sensitive and 21 as mechano-insensitive C-nociceptors (Type 1A and 1B of [40], respectively). Intracutaneous injection of IBA (2 μl of 1% IBA) with a 26 Gauge needle induced abundant bursts of ongoing spontaneous activity in 11 nociceptor units, all of them belonging to the mechano-insensitive class, which represents a 52.3% of all the recorded mechano-insensitive C-nociceptors (Figure 6). The time to activation of the units was variable, ranging from immediately after the injection (Figure 6B, unit in red), to several minutes after it (Figure 6B, units in blue and grey). Surprisingly, IBA injection did not activate any of the mechano-sensitive units, despite the fact that some of them had short-lasting response to the needle insertion, indicating that the injection site coincided with their receptive field (not illustrated). This finding is highly suggestive of a selective effect of IBA on the mechano-insensitive C-nociceptor class.

### Alkylamide effects on animal behavior

**Figure 7:**
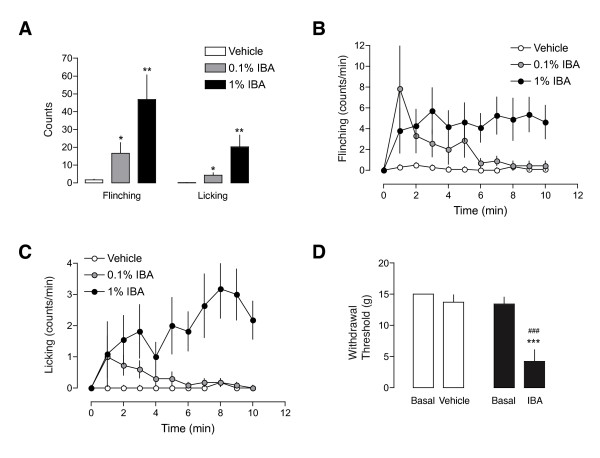
**Effects on alkylamide on pain behaviour**. **A**. Quantification of the number of flinching and licking behavior after intradermal injection of 2 ul IBA (0.1 or 1%) or vehicle in the rat hindpaw. The mean number of counts over a period of 10 min is shown (n = 11-14 animals per group). *p < 0.05; **p < 0.01 vs. vehicle injection. **B**. Time-course of flinching behavior. Number of counts per minute is shown for each group. IBA or vehicle was injected at time 0. **C**. Time-course of licking behavior. Number of counts per minute is shown for each group. **D**. Mechanical sensitivity to application of von Frey hairs. 50% mechanical threshold was measured using the up-down method before and after injection of vehicle or 1% IBA in the rat hindpaw (n = 8 animals in each group). ***p < 0.001 vs. basal threshold; ^###^p < 0.001 vs. vehicle injection.

Previous reports have shown that injection of IBA activates wide-dynamic range spinal neurons that receive convergent input from nociceptors thus implying that inhibition of K_2P _channels by alkylamides can trigger painful sensations [25]. In contrast, hydroxy-α-sanshool topically applied to the hindpaw failed to elicit any flinching or guarding behaviors [24]. Since our data shows that IBA activates a subset of small- and medium-sized sensory neurons that are thought to be involved in nociception, we next asked whether injection of IBA in the hindpaw evokes any nocifensive behavior. We injected 2 μl of 0.1% or 1% IBA and recorded the flinching and licking behavior during 10 min after the injection. At 0.1%, IBA produced a significant increase in flinching (16.6 ± 5.9 flinches; n = 14; p < 0.05) compared to vehicle injection (1.73 ± 0.5; n = 11; Figure 7A), showing a higher increase in the initial minutes after injection and declining towards baseline after 6 min (Figure 7B). The same concentration also produced a significant increase in licking behavior (4.4 ± 1.4 licks; n = 14; p < 0.05) compared with vehicle (0.18 ± 0.13; n = 11) and showed a similar temporal pattern. The effect was more robust with 1% IBA, with mean values of 46.9 ± 13.7 flinches (n = 11; p < 0.01; Figure 7A, B) and 20.3 ± 6.6 licks (n = 11; p < 0.01; Figure 7A, C). This time, the effect was more sustained and both flinching and licking behaviors lasted for more than 10 min (Figure 7B, C). Abundant guarding behavior was also observed during this time period (not shown).

In a different set of animals, threshold of evoked mechanical pain in response to von Frey hairs was measured before and 2 min after hindpaw injection of 1% IBA (Figure 7D). Vehicle injection did not produce any significant change in mechanical pain threshold (from 15 ± 0 to 13.7 ± 1.2 g; n = 8). In contrast, 1% IBA produced a marked decrease in mechanical pain sensitivity (13.4 ± 1.1 to 4.25 ± 1.8 g; n = 8), which was statistically significant compared with the basal value (p < 0.001) or with the control group (p < 0.001). All together, the results obtained suggest that peripheral blocking of background K^+ ^currents produces nocifensive behaviors in the animal.

### In vivo knock down of TRESK channels decreases threshold to painful mechanical stimuli

**Figure 8:**
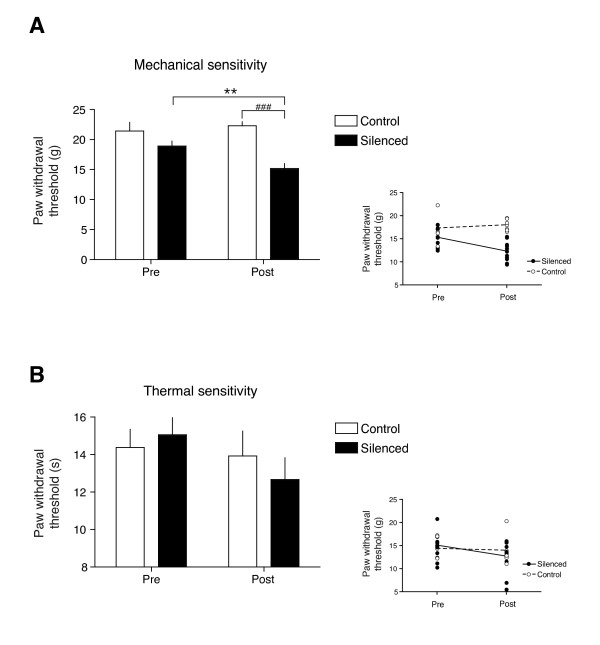
**TRESK silencing on painful sensitivity**. Paw withdrawal threshold in response to mechanical (**A**) or thermal (**B**) stimulation before (Pre) and after (Post) intrathecal siRNA injections. ** p < 0.01 vs. basal value in the silenced group (n = 10). ### p < 0.001 vs. control group (n = 6). Inset plots in Fig 8A and 8B show the change in mechanical or thermal thresholds in individual experiments. Dotted and solid lines represent the change in the mean value for control and silenced groups.

We used siRNA to reduce TRESK expression in lumbar DRG neurons in order to confirm the implication of TRESK channels in pain modulation. *In vivo *TRESK silencing led to a mRNA decrease in lumbar DRGs comparable to that found after axotomy (42.5% mean decrease in mRNA level; silenced vs. the group injected with control siRNA; data not shown; n = 10). When mechanical sensitivity was studied, paw withdrawal thresholds did not show significant differences in the baseline values (Pre) between groups. After siRNA injections, paw withdrawal threshold was unaltered in animals injected with the control siRNA (from 21.4 ± 1.5 to 22.3 ± 0.7 g; n = 6; Figure 8A). In TRESK-silenced animals, paw withdrawal threshold was significantly decreased (18.9 ± 0.8 to 15.1 ± 0.8; n = 10) when compared with the baseline level previous to siRNA injection (p < 0.01) or with the control group after control siRNA injection (p < 0.001). In contrast, paw withdrawal threshold to thermal stimulation did not significantly change neither in the control (from 14.4 ± 1.0 to 13.9 ± 1.3 s; Figure 8B) nor in the silenced group (15.0 ± 1.0 to 12.7 ± 1.2 s), despite a small tendency to decrease in the latter, suggesting that reduced TRESK expression may have a greater effect on the detection of pain in response to mechanical stimuli.

## Discussion

Sensory neurons display long-term hyperexcitability after crush or transection of their peripheral axons [7,9,10,41-44]. During the healing process, injury-induced hyperexcitability of primary afferent neurons is present until recovery of sensory axons and reinnervation of the peripheral target is achieved [8]. A variety of factors maintain this hyperexcitable state, which can become persistent and induce neuropathic pain in a proportion of patients [1,45]. Changes in the expression of several voltage-dependent channels contribute to the generation of hyperexcitability in sensory neurons, and particularly in nociceptors [6,46-53]. In contrast, how background conductances tune the excitability of sensory neurons is largely unknown. DRG and trigeminal neurons express several members of the K_2P _family of background K^+ ^channels [16,17,20], with TREK-1 being involved in polymodal pain perception [20], and the TREK/TRAAK family in heat and cold pain perception [21]. In small and medium-sized DRGs, many of which are nociceptors, TREK-2 and TRESK channels have a major contribution to total background current, while TREK-1 and TRAAK carry a smaller fraction of the current [18,19].

We have found that among those channels, only TRESK channels are down-regulated in response to injury both after *in vivo *or *in vitro *axotomy upon cell dissociation. This decrease in channel expression is well correlated with an increase in the injury marker ATF3 [33-37] and the α2δ1 subunit of the L-type Ca^2+ ^channel [30]. This is similar to what had been previously described in *Aplysia *nociceptors, where peripheral axon injury produces persistent hyperexcitability of nociceptive neurons and a reduction of the background S-type K^+ ^current [8,9]. Interestingly, this current, which shares similar pharmacological and electrophysiological properties with TREK-1 channels in mammals [12], contributes to sensory neuron hyperexcitability by increasing R_in _and decreasing rheobase current to AP firing [9]. Also, a decrease in the S-type K^+ ^current, which is persistently activated upon membrane depolarization (like TRESK), will favor repetitive firing of the neuron [8,9,18]. In contrast to the studies in *Aplysia *where recordings were performed in a semi-intact ganglia preparation, in the present study it was not possible to compare changes in background currents between neurons after axotomy due to the fact that acute dissociation also induces hyperexcitability in nociceptive neurons. Although we have not studied the time-dependence development of hyperexcitability in vitro after cell dissociation, this seems to appear quite early, since we could record hyperexcitable neurons as short as 3-4 h after plating. This observation has also been reported by others [11,31] and shows a good correlation with the expression of injury markers (ATF3 and Cacna2d1), changes in TRESK expression, or the lack of difference between background currents recorded in dissociated neurons (Figure 2) in conditions where most of the background current should be carried by TRESK (at room temperature other background channels are mostly inactivated; [19,32]). Interestingly, another study has reported up-regulation of TRESK expression in DRG neurons after several days in culture (Suppl. Fig S6 [54]), opening the possibility to down-regulation of TRESK channels after acute dissociation followed by recovery of their expression levels after several days in culture together with regenerative outgrowth of neurites.

Despite the proposed role of leak K^+ ^channels in setting membrane potential, we did not found differences in resting membrane potential after axotomy, which is in agreement with the lack of difference found in the resting membrane potential of DRG neurons from wild-type or TRESK[G339R] functional knockout mice [18]. This suggests that some compensation by other channels may be present in the knockout mouse or that TRESK has not a prominent role in setting resting membrane potential but on neuronal excitability. In fact, we observed an increase in TREK-1 expression in injured neurons compared with sham surgery (Figure 1B), which might compensate for TRESK reduction to maintain resting membrane potential. As mentioned, our electrophysiological recordings were done at room temperature, were some K_2P _channels appear to have a very low open probability [19]. If some compensation by other K_2P _channels was present, we might have underestimated their contribution when recording membrane potential or current, since it is possible that these channels were not active. On the other hand, it is also possible that low levels of TRESK expression after axotomy may be sufficient to maintain resting membrane potential but make the neuron more easily activated in response to depolarizing stimuli.

In this study we have used the sanshool derivative IBA which has been shown to elicit pungent burning, cooling and tingling sensations in humans [38]. IBA produces a transient depolarization of the resting membrane potential that is sufficient to activate the DRG neuron and induce Ca^2+ ^entry (Figure 5A), as proposed for hydroxy-α-sanshool [23] and IBA [55]. It is possible that the depolarization found in vitro after IBA application (~40 mV) may be larger than in physiological conditions due to the downregulation of TRESK after neuronal dissociation. Nevertheless, the effects found in vivo (Figure 6, 7) as well as recently reported data [55], suggested that even if TRESK is normally expressed, the block elicited by IBA is sufficient to depolarize the neuron and induce neuronal firing.

Despite not being completely selective for TRESK channels (Figure 5B), it seems that the major action of IBA is due to the blocking effect on this channel since an overall block of the K^+ ^current can be seen in native DRGs (Figure 4E, F). It has been suggested that hydroxy-α-sanshool and, by extension IBA, could activate other channels such as TRPV1 or TRPA1 [54,56]. In contrast, others have discarded this effect from studies on knockout mice [23]. Our study and a recent characterization of IBA effects on DRG neurons [55] show that this compound activates different subsets of neurons, some of them expressing TRPV1, TRPA1 or TRPM8, but also some neurons not responding to well-known agonists of these TRP channels. Therefore, it seems that effects of IBA are mainly mediated by inhibition of K_2P _channels although it can't be completely ruled out that IBA does some unidentified effect on intracellular calcium signaling or on TRPs. A detailed study on IBA selectivity remains to be performed.

In this study, most IBA-sensitive neurons were in the small- and medium-size range and about 70% of them responded to capsaicin. Therefore, it is likely that most of those neurons were unmyelinated nociceptors. The other 30% only responded to IBA, but not to capsaicin, probably representing either the fraction of neurons with slowly conducting C-fibers insensitive to capsaicin or D-hair fibers (Aδ). Large DRGs activated by IBA probably correspond to large myelinated sensory afferents with Aβ axons [23,24]. In agreement to these observations, the important biological role of TRESK is further demonstrated by the potent activation of peripheral C-nociceptor units *in vivo *after IBA injection in the rat paw. This is consistent with the effects of hydroxy-α-sanshool on the skin-nerve preparation [24] or peripherally applied IBA on low threshold mechanosensitive neurons and in wide dynamic range type neurons, that receive input from mechanoreceptors and nociceptors [25]. IBA-induced activation seemed to occur in a particular class of peripheral C-nociceptors, namely the mechano-insenstive ones, but not in the mechano-sensitive ones. The majority of mechano-insensitive C-nociceptors are peptidergic, NGF-dependent, IB4-negative peripheral nociceptors, which have been recently shown to have an important role in neuropathic pain conditions [57,58]. Our findings suggest that background currents mediated by TRESK may be important in this specific class of peripheral nociceptors. Blockage of TRESK channels *in vivo *not only induced spontaneous activity in C-nociceptors, but also resulted in a behavioral sensitization to mechanical stimuli. The decrease in the threshold for evoked mechanical pain after IBA injection or TRESK knockdown, opens the possibility that C-fibers that are mechanically insensitive in normal conditions, became sensitive after decreasing the total amount of background current. Despite the apparent paradox that pain and hyperalgesia to mechanical stimulation are encoded by mechano-insensitive nociceptors, mechanical sensitivity of previously mechanically-insensitive C-fibers have been already reported due to sensitization by capsaicin [59] or tonic pressure [60]. Although the cellular mechanisms underling these changes are still unknown, different possibilities exist, like unmasking of stretch-activated membrane channels, release of chemical mediators generated by mechanical stimulation or a decrease/block of a K^+ ^conductance (e.g. TRESK), which will make mechanical stimulation more effective to activate the fiber.

Injection of IBA in the rat hindpaw produced a dose-dependent nocifensive behavior that shows a good correlation with the effects of this compound in cultured sensory neurons, in the activation of sciatic nerve C-fibers and with recently reported results [55]. Consistent with these effects, sanshool-containing water produced aversion in mice [23] and burning sensation in humans [38]. In contrast, another study failed to demonstrate any nocifensive behavior after topical application of sanshool to the rat hindpaw [24]. Skin penetration of sanshool after topical application may not be sufficient to reach and activate nociceptor terminals, but direct drug injection in the paw is able to activate them, like in reports by Sawyer et al. [25], Klein et al. [55] and in the present study. Our finding that knocking down TRESK expression decreases the threshold to mechanical painful stimuli is also consistent with the effects found on animal behavior and to the suggested involvement of TRESK in mediating tingling paresthesia [24,38], therefore implicating TRESK channels in pain sensation. The apparent selectivity of TRESK silencing on mechanical but not heat thresholds is difficult to rationalize with the present findings, but could be due to an incomplete knock down of TRESK expression. We cannot rule out that effects on thermal painful perception will appear with higher levels of silencing or by completely knocking out the channel expression. Similarly, a decrease in mechanical withdrawal threshold but not in heat withdrawal latency after IBA injection has been reported [55]. In addition, a recent report shows only a slight increase in thermal nociceptive sensitivity (20% decrease in latency in the hot plate test) in TRESK knockout mice [61]. A detailed study on the role of this ion channel in different sensory modalities should come from further analysis of this TRESK-deficient mouse.

The regulation of TRESK currents after injury shown here suggests a possible role of this channel in the generation of allodynia and/or hyperalgesia caused by nerve injury. Blocking or silencing the channel we also show that TRESK participates in nociceptor excitability and behavioral responsiveness in normally behaving animals, but the role of TRESK in pathological conditions (after injury or in different pain models) remains to be further investigated. TRESK is particularly interesting since it is the only background channel activated by an increase in intracellular Ca^2+ ^[15,62], a common signaling mechanism found after activation of nociceptors by many compounds. Between resting membrane potential and spike threshold, a decrease in TRESK currents may be critical for opposing depolarizing inputs, as other major outward currents are inactivated (except background currents), outside the voltage range for effective activation, or relatively inactive in the absence of Ca^2+ ^influx that occurs during action potentials. This is in general agreement with the results from the TRESK[G339R] functional knockout mice [18] or the recently reported association of a dominant-negative mutation in the human channel in certain cases of familial migraine with aura [22]. A decrease in TRESK functionality may also underlie the appearance of CIPS (Cyclosporine-Induced Pain Syndrome) due to the use of calcineurin inhibitors (cyclosporine; FK506) [63,64] or the increase in the anesthetic isoflurane (a TRESK activator) requirement after cyclosporine treatment [65]. Because inhibiting calcineurin will impair TRESK activation in response to stimuli-induced Ca^2+ ^increase, a higher requirement of this volatile anesthetic will be needed to achieve anesthesia, as recently shown in the knockout mice [61].

## Conclusions

In summary, we show that axotomy downregulates TRESK expression, which may contribute to enhanced excitability after nerve injury. In good agreement, we demonstrate that in normally behaving animals, pharmacological inhibition of channel activity or siRNA silencing in nociceptors increase pain sensitivity and painful animal behavior, supporting an important role for TRESK in nociceptor excitability.

## Methods

### Animal surgery

All experimental procedures were carried out in accordance with the recommendations of the International Association for the Study of Pain (IASP) and were reviewed and approved by the University of Barcelona Animal Care Committee (Ref. 5336, 5406). Adult male Sprague-Dawley rats (Harlan; 100-150 g) were kept at 22°C with free access to food and water in an alternating 12 h light and dark cycle. Rats were anesthetized with isoflurane and a small incision in the skin was made to separate the muscle and expose the sciatic nerve, that was transected proximal to the bifurcation into the tibial and peroneal divisions as previously described [6,7]. To avoid nerve regeneration, a 3 mm segment of the nerve was removed. The same procedure was performed in sham animals without transecting the nerve. To prevent foot mutilation, Mordex^® ^(Lab. URGO, Hernani, Spain) was applied to the operated foot. Daily inspections on operated animals were done to observe possible autotomy, which was scored according to the scale described by Wall et al. [66]. None of the animals used in the study attained a score of more than 5. After surgery, animals were kept for 3 weeks to allow the development of neuronal hyperexcitability due to axotomy. After this period, animals were anesthetized with isofluorane, killed by decapitation and DRGs (L4 and L5) from injured and contralateral uninjured sides were removed for neuronal culture or for RNA extraction.

### DRG neuron culture

L4 and L5 DRG were collected in cold phosphate buffered saline (PBS) with glucose, cleaned with iridectomy scissors under an stereoscopic microscope and incubated in phosphate buffered saline (PBS, Sigma) supplemented with 10 mM glucose, 10 mM Hepes, 100 U.I./mL penicillin, 100 μg/mL streptomycin, and collagenase type IA (4-5 mg/ml, Sigma) for 60 min at 37°C with gentle shaking. Digested ganglions were gently triturated with head-polished Pasteur pipettes; collagenase was inhibited by adding the solution to 10 ml of Dulbecco's Modified Eagle's Medium (DMEM) containing 10% fetal bovine serum and the mixture was centrifuged at 1000 rpm for 5 min. The pellet was suspended in DMEM plus 10% fetal bovine serum, 100 mg/mL L-glutamine, 100 U.I./mL penicillin, 100 μg/mL streptomycin and the suspension was plated on glass coverslips treated with poly-L-lysine/laminin and placed in culture dishes in an incubator at 37°C and 95% air, 5%CO_2_. No NGF or other growth factors were added. Cells were used for electrophysiological recording within 3-48 h of plating.

### Calcium imaging

DRG neurons obtained as described above were plated on 25 mm diameter glass coverslips (VWR Scientific Inc., Philadelphia, PA) and used 24-48 h thereafter. Cells were loaded with 5 μM fura-2/AM (Calbiochem, San Diego, CA) for 45-60 min at 37°C in incubation buffer (140 mM NaCl, 4.3 mM KCl, 1.3 mM CaCl_2_, 1 mM MgCl_2_, 10 mM glucose, 10 mM HEPES, at pH 7.4 with NaOH). Coverslips with fura-2 loaded cells were transferred into an open flow chamber (1 ml incubation buffer) mounted on the heated stage of an inverted Olympus IX70 microscope using a TILL monocromator as a source of illumination. Pictures were taken with an attached cooled CCD camera (Orca II-ER, Hamamatsu Photonics, Japan) and were digitized, stored and analyzed on a PC computer using Aquacosmos software (Hamamatsu Photonics, Shizuoka, Japan). After a stabilization period, image pairs were obtained alternately every 4 s at excitation wavelengths of 340 (λ1) and 380 nm (λ2; 10 nm bandwidth filters) in order to excite the Ca^2+ ^bound and Ca^2+ ^free forms of this ratiometric dye, respectively. The emission wavelength was 510 nm (120-nm bandwidth filter). Typically, 5-10 cells were present in a field and [Ca^2+^]_i _values were calculated and analyzed individually for each single cell from the 340- to 380-nm fluorescence ratios at each time point. Several experiments with cells from different primary cultures were used in all the groups assayed.

### RNA extraction and Quantitative real-time PCR

For each animal, RNA from L4-L5 DRG pairs (axotomized and contralateral control) was extracted with Trizol (Sigma, Madrid) and first-strand cDNA was transcribed using the RETROscript kit (Ambion). qPCR experiments were performed in an AbiPrism 7300 using the TaqMan Universal PCR MasterMix (Applied Biosystems) with primers obtained from TaqMan Gene Expression assays: Rn00597042_m1 (TREK-1); Rn00576558_m1 (TREK-2); Rn00583727_m1 (TASK-1); Rn00587450_m1 (TRAAK); Rn99999916_s1 (GADPH); Rn00563784_m1 (ATF3); Rn00563853_m1 (CaCna2d1). A Custom Taqman Gene expression assay (Applied Biosystems) was designed for rat TRESK using the following primers: forward: TGCACAGTGTTCAGCACAGT; Reverse: CATATAGCATGCACAGGAACTTACC. Amplification of GADPH transcripts was used as a standard for normalization of all qPCR experiments and gene fold-expression was assessed with the ΔΔC_T _method ipsilateral vs. contralateral side. Experiments were performed in quadruplicate.

### Electrophysiological recording

Electrophysiological recordings were performed with a patch-clamp amplifier (Axopatch 200B, Molecular Devices, Union City, CA) and restricted to small and medium DRG neurons (<30 μm; <45 pF), which largely correspond to nociceptive neurons. Patch electrodes were fabricated in a Flaming/Brown micropipette puller P-97 (Sutter instruments). Electrodes had a resistance between 4-7 MΩ when filled with intracellular solution (in mM): 97.5 K^+^-gluconate, 32.5 KCl, 1 MgCl_2_, 5 EGTA, 10 HEPES at pH 7.2 and 300 mOsm/Kg. An artificial cerebrospinal fluid (ACSF) was used as bath (in mM): 125 NaCl, 2.5 KCl, 0.5 CaCl_2_, 2.5 MgCl_2_, 1.25 NaH_2_PO_4_, 26 NaHCO_3_, 10 glucose, 2 Na-piruvate, 3 myo-inositol, 0.5 ascorbic acid at pH 7.4 and 310 mOsm/Kg. Membrane currents were recorded in the whole-cell patch clamp configuration, filtered at 2 kHz, digitized at 10 kHz and acquired with pClamp 9 software and in the presence of 2 μM TTX. Data was analyzed with Clampfit 9 (Molecular Devices) and Prism 4 (GraphPad Software, Inc., La Jolla, CA). Series resistance was always kept below 30 MΩ and compensated at 70-80%. All recordings were done at room temperature (22-23°C). When studying the excitability of neurons in culture, after achieving the whole-cell configuration, the amplifier was switched to current-clamp bridge mode. Only neurons with a resting membrane voltage below -50 mV were considered for the study. To study neuronal excitability, we examined the resting membrane potential (RMP); action potential (AP) current threshold elicited by 20 ms depolarizing current pulses in 0.05-0.1 nA increments; whole-cell input resistance (R_in_) was calculated on the basis of the steady-state I-V relationship during a series of 100-ms hyperpolarizing currents delivered in steps of 0.01-0.02 nA from 0.2 to 0.1 nA; AP amplitude (measured from RMP to AP peak; AP duration (measured at 50% AP amplitude); AHP (measured from RMP to peak hyperpolarization). Repetitive discharge was measured by counting the spikes evoked by 1-s, intracellular pulses of depolarizing current normalized to 2.5 times the AP threshold current. In some experiments (Figure 5A), calcium imaging and recording of membrane voltage was simultaneously performed. Cells were loaded first with fura-2 as described previously. Next, the whole-cell patch clamp configuration was achieved and the amplifier was switched to current-clamp bridge mode to record membrane voltage. Despite a slow decrease of fura-2 fluorescence values due to cell content dialysis, the short duration of the recording and the ratiometric measurement with fura-2 compensated for this effect.

### Recordings in the excised intact ganglion

Intracellular recordings in the excised ganglion were performed using an Axoclamp2B amplifier (Molecular Devices, Union City, CA) in the bridge-mode configuration. Axotomized or control intact L4 or L5 ganglia were treated with collagenase IA 4 mg/ml for 30 min at 37°C and then transferred to a recording chamber mounted in the stage of an upright BX50-WI microscope (Olympus, Japan). The ganglion was fixed with a nylon mesh that allowed the passage of the recording electrode through the mesh fibers. Pipettes filled with 3M K^+^-acetate had a resistance of 80-130 MΩ. Recordings were performed in the ganglion bathed in artificial cerebrospinal fluid (ACSF) solution at room temperature (22-23°C). Only neurons that had a resting membrane potential below -50 mV and a R_in _of more than 50 MΩ were included in the study.

### Electrophysiology in transfected cells

HEK293T cells cultured in DMEM with 10% FBS were seeded in 35-mm dish 24 h before transfection. Cells were transiently transfected with pEGFP vector alone (control) or cotransfected with: rTRESK-pcDNA3.1 (kindly provided by Dr. S. Yost, University of California-San Francisco), pCD8-mTREK-1, pCD8-hTREK-2 or pCD8-mTRAAK (kindly provided by Dr. F. Lesage, Institut de Pharmacologie Moléculaire et Cellulaire-CNRS, Valbonne, France) using FuGene transfection reagent (Roche). Transfected cells were used for electrophysiological recordings 24-48 h after. Patch clamp recordings were performed as described above. For whole-cell experiments, the solutions used were as follows. Intracellular solution (in mM): 140 KCl, 2.1 CaCl_2_, 2.5 MgCl_2_, 5 EGTA, 10 HEPES at pH 7.3. Bath solution (in mM): 145 NaCl, 5 KCl, 2 CaCl_2_, 2 MgCl_2_, 10 HEPES at pH 7.4. Cells were continuously superfused with a microperfusion system during the experiments, which were done at room temperature. When studying the membrane potential, after achieving the whole-cell configuration, the amplifier was switched to current-clamp bridge mode.

### Flinch test

Rats were injected with 2 μl of a solution containing 0.1 or 1% IBA or propylene glycol (control vehicle) administered intradermally in the hindpaw using a 10 μl, 26 g Hamilton syringe. The rat behavior was observed and the number of flinching and licking of the paw was recorded every minute for a 10 min period starting immediately after the injection.

### Mechanical sensitivity

To assess mechanical sensitivity, the withdrawal threshold to punctate mechanical stimuli of the hindpaw was determined before and 2 min after 1% IBA injection in the hindpaw (as previously described) by the application of calibrated von Frey filaments (North Coast Medical, Inc. Morgan Hill, CA). The von Frey filaments [3.92, 5.88, 9.80, 19.60, 39.21, 58.82, 78.43, and 147.05 mN; equivalent to (in grams) 0.4, 0.6, 1, 2, 4, 6, 8, and 15] were applied vertically to the plantar surface of the hindpaw and gently pushed to the bending point. The 50% withdrawal threshold was determined using the up-down method as previously described [67]. A brisk hindpaw lift in response to von Frey filament stimulation was regarded as a withdrawal response.

### Microneurographic recordings

Microneurographic recordings were obtained from 6 Spague-Dawley male rats (weight 200-250 g) anesthetized with ketamine (90 mg/kg) and xylacine (10 mg/kg) injected intraperitoneally. The sciatic nerve was exposed at mid-thigh level and intraneural recordings were performed according to the method recently described in detail elsewhere [57]. In brief, tungsten microelectrodes (200 μm diameter, lacquer-insulated, nominal impedance 1MΩ) were inserted into the sciatic nerve trunk with the aid of a micromanipulator. A subcutaneous reference electrode was inserted outside the nerve trunk. The neural signals were amplified with an isolated, high input impedance amplifier (3+ MicroAmp, FHC, USA), bandpass filtered (maximum range 50-5,000 Hz) and fed to a noise eliminator (Hum Bug, Quest Scientific, North Vancouver, Canada). This signal was then fed to a digital audio-monitor (AM10 audio monitor, Grass Technologies, Astro-Med, Inc., USA). Temperature of the skin was measured with a thermocouple placed on the skin adjacent to the receptive fields of the units under study. Electrical stimuli were triggered, and the responses to electrical stimulation recorded and analyzed with a PC and data acquisition board (National Instruments, PCI-6221, USA). The digitized responses were stored on the hard drive of the PC as raw data for offline analysis. Digital filtering (band pass 0.3-2 kHz) and clamping of the baseline were performed both on-line and during off-line analysis for a better visualization of the action potentials.

Responses were recorded with QTRAC software (^©^Institute of Neurology, London, UK), using the facility to determine multiple peak latencies and display them as latency "profile" or raster plot. In the latency raster plots, each peak in the filtered voltage signal that exceeded a specified level is represented by a dot on a plot with latency as the ordinate and elapsed time as the abscissa. Depending on the level chosen, the dots could represent action potentials or noise. For the raster figures shown in this paper, latencies of selected units with adequate signal-to-noise were remeasured from the raw data, so that each dot represents an identified single unit. These remeasured figures are referred to as "modified" raster plots. Activity-dependent slowing of C fibers was assessed using the protocol described by Serra et al. [40,68]. This consists of a sequence of: 1) baseline stimulation at 0.25 Hz for 3 minutes; 2) 3-min pause; 3) 6-min at 0.25 Hz; 4) 3-min 2 Hz train; 5) return to 0.25 Hz baseline until the latencies return to their original values (see Figure 6). This method differentiates "profiles" of activity-dependent slowing in individual C fibers of humans and rats that correspond to specific functional types of peripheral neurons [40,68-70]. Fibers slowing more than 10% during 3-min 2 Hz trains are nociceptors (Type 1 fibers in [68]). Further classification of C-nociceptor type into mechano-sensitive and mechano-insensitive units was achieved by studying the degree of slowing at very low frequencies after a pause [40,71].

### In vivo injection of siRNA and behavioural tests

siRNA targeting rat TRESK (s175514; Sense AGAGAUUGGUUGCUCGAGAtt; Antisense UCUCGAGCAACCAAUCUCUca) and siRNA Negative Control #1 (4390843) were purchased from Applied Biosystems (Silencer^® ^Select Pre-designed siRNA) and injected in rats by intrathecal bolus to the lumbar region of the spinal cord once a day for 3 days. Each 10-μl injection corresponded to 2 μg of siRNA complexed with in vivo-jetPEI transfection reagent (Polyplus-transfection SA, Illkirch, France) following the supplier's suggested protocol. The specificity of the effect was evaluated in lumbar dorsal root ganglia by qPCR. On the same day of the behavioral tests (24 h after the last siRNA injection) L4 and L5 ganglia were removed and immediately frozen in liquid N_2_. Later, RNA was extracted, cDNA was prepared and used for qPCR using the same primers and following the procedure previously described.

Heat sensitivity of adult male Sprague-Dawley rats (Harlan) was assessed by measuring hindpaw withdrawal latency from radiant infrared source (Hargreaves Method) using a Ugo Basile (Italy) Model 37370 Plantar test. Rats were acclimated to the experience room for at least 30 min and each measurement was the mean of 5 trials. Withdrawal latency was measured 24 h before and 24 h after the last intrathecal injection. Mechanical sensitivity was assessed by measuring hindpaw withdrawal latency with a Dynamic Plantar Aesthesiometer (37450; Ugo Basile, Italy). Incremental force (0-50 g in 40 s ramp) was applied with a 2 mm diameter metal rod to the paw plantar side. When the rat withdrew its paw, mechanical stimulus stopped automatically and time (s) and force (weight in grams) of paw withdrawal was recorded. Paw withdrawal responses were the average of 5 measures. The same experimenter for a given test performed all behavioral experiments, which was blind to the treatment applied to the animal. All measurements were done in a quiet room, taking great care to minimize or avoid discomfort of the animals.

### Drugs

Isobutylalkenyl amide (IBA) was kindly provided by Givaudan (Cincinnati, OH) and initially diluted in dimethylformamide (DMF) at a 100 mM. For in vitro experiments, IBA stock was subsequently diluted in the appropriate medium and used at the stated concentrations (100-500 μM), similarly to what has been reported for hydroxy-α-sanshool [23]. Final concentration of DMF was 0.1% or below. For in vivo experiments (hindpaw injections), IBA was diluted in propylene glycol and used at 1 or 0.1% as previously reported [25]. Lamotrigine, tetrodotoxin (TTX) and capsaicine (Cap) were purchased from Sigma (Madrid, Spain).

## List of abbreviations

DRG: dorsal root ganglion; IBA: Isobutylalkenyl amide; R_in_: input resistance; AP: action potential; RMP: resting membrane potential; AHP: afterhyperpolarization; TTX: tetrodotoxin; eGFP: enhanced green fluorescent protein; DMF: dimethylformamide.

## Competing interests

The authors declare that they have no competing interests.

## Authors' contributions

Authors AT, XG performed animal surgery, quantitative PCR, electrophysiological recordings and calcium imaging. AT, XG and GC performed behavioral experiments. AT, GC, BS carried out cellular cultures, plasmid generation and transfection. BC and JS performed microneurography experiments. AT, JS and XG participated in the design of the study and performed the statistical analysis. XG conceived of the study, oversaw the research and prepared the manuscript with help from all others. All authors read and approved the final manuscript.
